# Subthreshold IKK activation modulates the effector functions of primary mast cells and allows specific targeting of transformed mast cells

**DOI:** 10.18632/oncotarget.3022

**Published:** 2015-02-27

**Authors:** Sebastian Drube, Franziska Weber, Romy Loschinski, Mandy Beyer, Mandy Rothe, Anja Rabenhorst, Christiane Göpfert, Isabel Meininger, Michaela A. Diamanti, David Stegner, Norman Häfner, Martin Böttcher, Kirstin Reinecke, Thomas Herdegen, Florian R. Greten, Bernhard Nieswandt, Karin Hartmann, Oliver H. Krämer, Thomas Kamradt

**Affiliations:** ^1^ Institut für Immunologie, Universitätsklinikum Jena, 07743 Jena, Germany; ^2^ Klinik und Poliklinik für Dermatologie und Venerologie, Universität zu Köln, 50937 Köln, Germany; ^3^ Georg-Speyer-Haus, Institute for Tumorbiology and Experimental Therapy, 60596 Frankfurt, Germany; ^4^ Rudolf Virchow Centrum für experimentelle Biomedizin, Universität Würzburg, 97080 Würzburg, Germany; ^5^ Gynäkologische Molekularbiologie, Klinik für Frauenheilkunde und Geburtshilfe, 07743 Jena, Germany; ^6^ Institut für Experimentelle und Klinische Pharmakologie, Universität Schleswig-Holstein, 24105 Kiel, Germany; ^7^ Institut für Toxikologie, Universitätsmedizin Mainz, 55131 Mainz, Germany

**Keywords:** Mast cells, subthreshold IKK activation, mitogenic signaling, NFκB-activation

## Abstract

Mast cell differentiation and proliferation depends on IL-3. IL-3 induces the activation of MAP-kinases and STATs and consequently induces proliferation and survival. Dysregulation of IL-3 signaling pathways also contribute to inflammation and tumorigenesis. We show here that IL-3 induces a SFK- and Ca^2+^-dependent activation of the inhibitor of κB kinases 2 (IKK2) which results in mast cell proliferation and survival but does not induce IκBα-degradation and NFκB activation. Therefore we propose the term “subthreshold IKK activation”.

This subthreshold IKK activation also primes mast cells for enhanced responsiveness to IL-33R signaling. Consequently, co-stimulation with IL-3 and IL-33 increases IKK activation and massively enhances cytokine production induced by IL-33.

We further reveal that in neoplastic mast cells expressing constitutively active Ras, subthreshold IKK activation is associated with uncontrolled proliferation. Consequently, pharmacological IKK inhibition reduces tumor growth selectively by inducing apoptosis *in vivo*.

Together, subthreshold IKK activation is crucial to mediate the full IL-33-induced effector functions in primary mast cells and to mediate uncontrolled proliferation of neoplastic mast cells. Thus, IKK2 is a new molecularly defined target structure.

## INTRODUCTION

Mast cells are located in peripheral tissues and regulate innate and adaptive immune responses [1] by producing mediators (e.g., histamine, proteases, leukotrienes or cytokines) that recruit and activate, granulocytes, dendritic cells, T-lymphocytes and other cells [1–5]. They are critical in type I hypersensitivity and therefore central to the pathogenesis of allergic diseases [6]. The participation of mast cells in the pathogenesis of autoimmune diseases has been reported [7] and refuted [8]. Mast cells can produce pathogenetically relevant cytokines such as IL-1β, IL-6, IL-13, IL-17 and TNFα [9]. It has also been demonstrated that mast cells are critical regulators of the tumor microenvironment [10] and that expression of constitutively active Ras- or c-Kit-mutants leads to development of mast cell tumors [11, 12].

The most extensively characterized trigger for mast cell activation is crosslinking of the Fcε-receptor-I (FcεRI) resulting in the release of mediators including histamine, proteases, cytokines, and chemokines [13].

Mast cells express TLR/interleukin-1 (IL-1) receptor (TIR) family members including TLR4 and the IL-33-receptor (IL-33R) [1, 14]. IL-33 induces antigen-independent activation of mast cells [15] via the canonical NFκB signaling [16] resulting in cytokine production but not degranulation [15].

In contrast, IL-3 is crucial to promote differentiation, survival, and proliferation of mast cells [17–19]. IL-3 activates STAT3/5, JNK1/2, ERK1/2, PI3Ks and mTOR [17, 20, 21]. Impairment of these signaling cascades decreases mast cell numbers in peripheral tissues, compromises bacterial clearance and weakens type I hypersensitivity [17, 20, 22–24].

Recent publications reported a crosstalk between FcεRI either with TLRs (including TLR4) or c-Kit resulting in increased NFκB-, NFAT- or JNK activation and cytokine production [25, 26]. We found an crosstalk between activated c-Kit and the IL-1R or the IL-33R [27, 28] resulting in potentiated cytokine production in response to IL-1 or IL-33. Here, we identify a novel mechanism by which IL-3 induces IKK activation which mediates mitogenic signaling in primary- and tumor mast cells and modulates the full biological response of IL-33.

## RESULTS

### IL-3-induced IKK activation in BMMC

**Figure 1:**
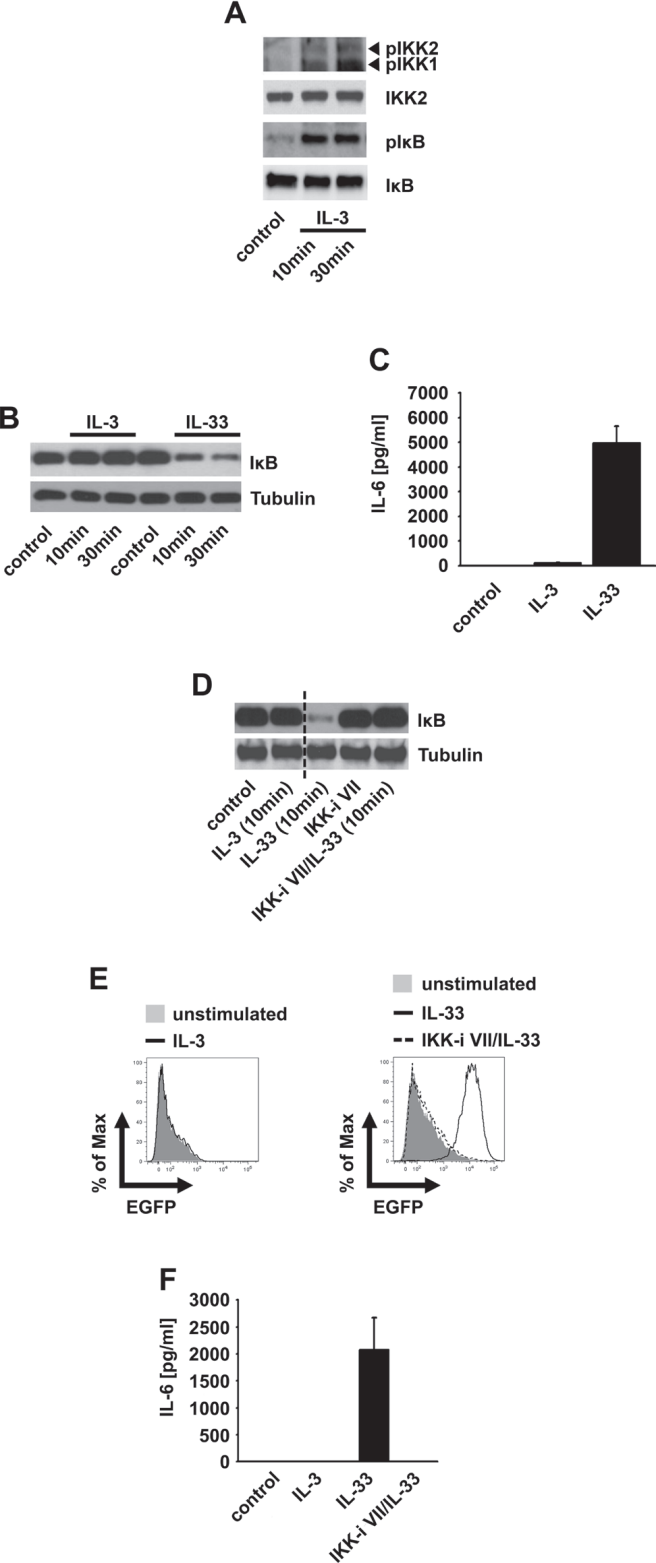
The IL3-induced IKK activation does not mediate IκBα degradation **(A, B)** BMMCs were stimulated with IL-3 **(A, B)** or IL-33 **(B)** Lysates were analyzed by westernblotting. **(C)** BMMCs were stimulated with IL-3 or IL-33. Supernatants were collected and analyzed for IL-6. **(D–F)** NFκB-EGFP-MC/9 cells were pre-incubated with the IKK-inhibitor VII and stimulated with IL-3 or IL-33. Lysates were analyzed by westernblotting **(D)** or cells were analyzed for EGFP-production by flow cytometry **(E)** or collected supernatants were analyzed for IL-6 **(F)**

IL-3 induced IKK activation results in anti-apoptotic signaling in hepatocytes [29]. We found that IL-3 also induces IKK activation, IκBα phosphorylation but not degradation in BMMCs (Figure 1A). To exclude defective IKK-IκBα signaling we used IL-33, a known activator of IKK-dependent IκBα degradation. In contrast to IL-3, IL-33 induced IκBα degradation and cytokine response in BMMCs which was blocked by treatment with the IKK-inhibitor VII (Figure 1B and 1C), the most efficient IKK inhibitor tested (Supplementary Figure S1A–D).

Next we used NFκB-EGFP-MC/9 [30] mast cells to examine whether IL-3 induces NFκB activation. Confirming the results in BMMCs, IL-3 did not induce IκBα degradation (Figure 1D), EGFP expression (Figure 1E) or an effective cytokine-production (Figure 1F). Again, IL-33 elicited all these effects in an IKK-dependent manner (Figure 1D–1F). Thus, IL-3 does not induce the canonical NFκB signaling. Why does the IL-3-induced IKK activation fail to activate NFκB? When cells were cultured with cycloheximide, an inhibitor of protein biosynthesis, IL-3 induced a mild but detectable IκBα degradation (Supplementary Figure S1E). This finding indicates that IL-3 can induce some IκBα degradation. In presence of ongoing IκBα re-synthesis, this IL-3-induced IκBα degradation is quantitatively not sufficient to result a net loss of IκBα. Therefore, we propose the term “subthreshold activation” to describe IKK activation which suffices to phosphorylate IκBα but is insufficient to trigger NFκB activation.

### Subthreshold IKK2 activation is critical for IL-3-induced proliferation

**Figure 2:**
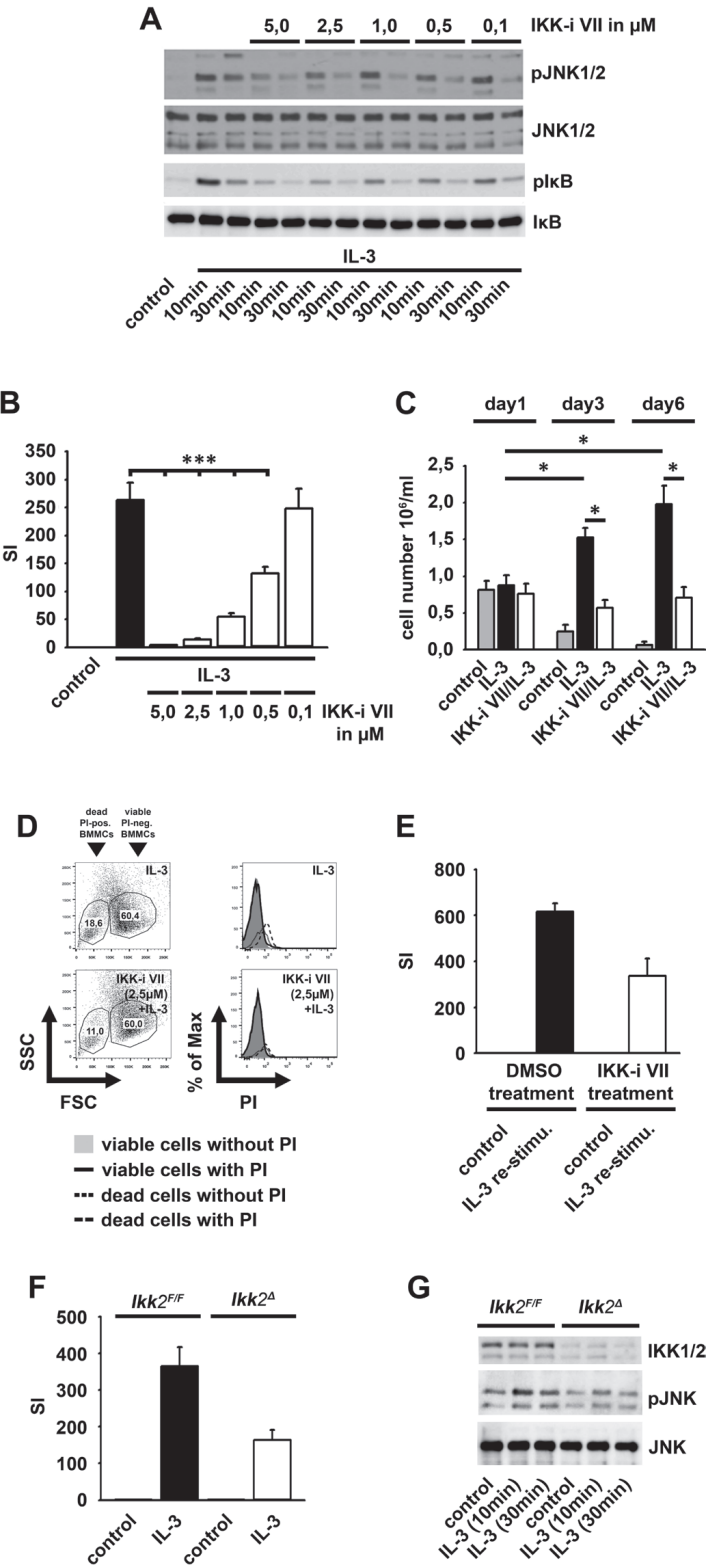
The IL-3-induced IKK2 activation mediates mitogenic signaling **(A, B)** BMMCs were pre-incubated with the IKK-inhibitor VII and were stimulated with IL-3. Lysates were analyzed by westernblotting **(A)** or cells were probed with [H^3^]thymidine and analyzed by *β*-counting **(B)** (*p* < 0,001). **(C, D)** BMMCs were pre-treated with the IKK-inhibitor VII (2,5 μM) and stimulated with IL-3. BMMCs were treated with toloduine blue **(C)** or PI **(D)** and viable cells were counted **(C)** (*p* < 0,01) or cells were analyzed by flow cytometry **(D)**. **(E)** IKK-inhibitor VII-treated (2,5 μM) BMMCs were cultured with IL-3 (for 48 h), washed, re-stimulated with IL-3 (for 4 days), probed with [H^3^]thymidine and analyzed by *β*-counting. **(F, G)** *Ikk2^F/F^* or *Ikk2^Δ^* BMMCs were stimulated with IL-3. Cells were probed with [H^3^]thymidine and analyzed by *β*-counting **(F)** (*p* < 0,001) or lysates were analyzed by westernblotting **(G)**.

Having shown that the IL-3-induced subthreshold IKK activation does not result in efficient cytokine production, we asked if IKKs mediate the activation of the mitogenic JNK signaling [24]. Indeed, the IKK-inhibitor VII potently impaired the IL-3-induced activation of JNKs and the phosphorylation of IκBα (Figure 2A). Consequently, the IKK-inhibitor VII blocked the IL-3-induced proliferation (Figure 2B) and BMMC expansion (Figure 2C) without inducing cell death (Figure 2D). When IKK-inhibitor VII-treated BMMC were washed and re-stimulated with IL-3, their proliferative capacity was restored (Figure 2E).

To confirm the results obtained by pharmacological inhibition of IKKs, we induced IKK2-deficiency by injection of *Ikk2^Δ^*-mice with poly(I:C) [31]. Consequently, the IL-3-induced proliferation and JNK activation was reduced in *Ikk2^Δ^*-BMMC compared to *Ikk2^F/F^*-BMMCs (Figure 2F and 2G).

### The IL-3-induced proliferation depends on JNK1

**Figure 3:**
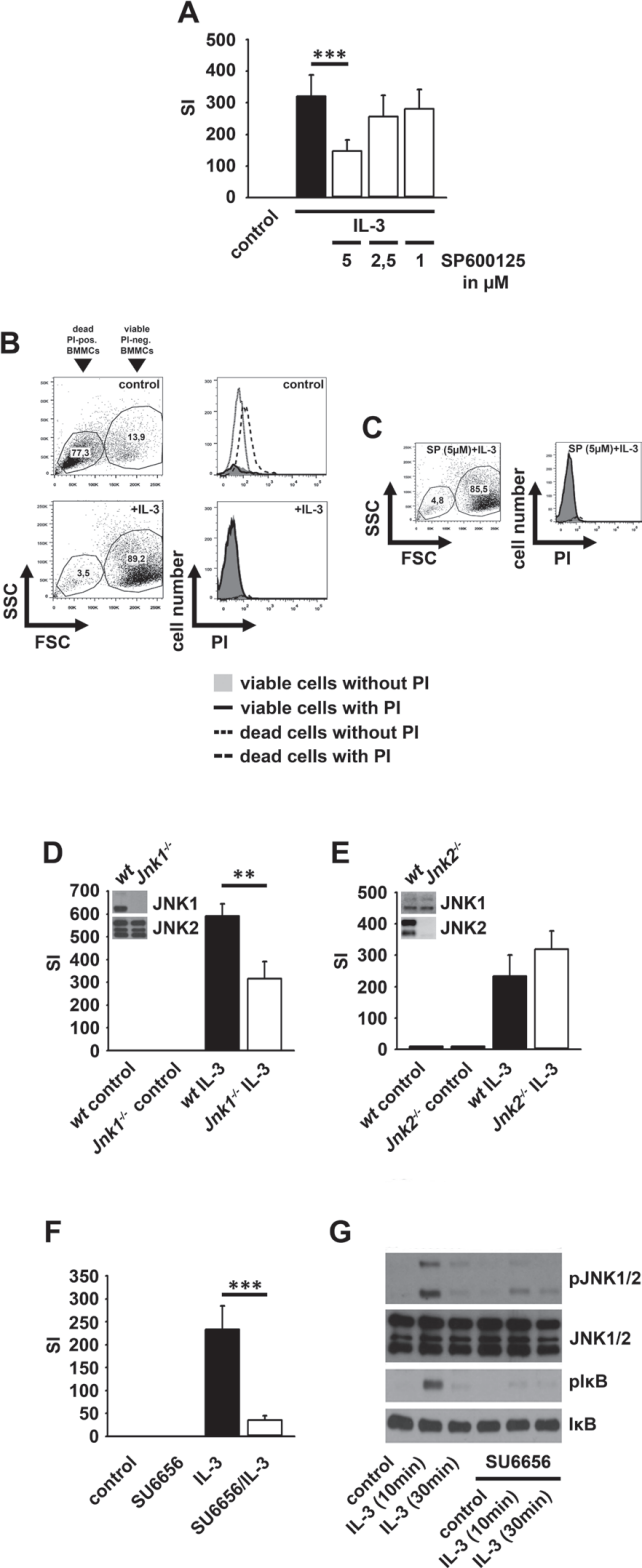
JNK1 and SFKs mediate the IL-3-induced mitogenic signaling **(A–C)** BMMCs were pre-treated with SP600125. Cells were probed with [H^3^]thymidine **(A)** or PI **(B, C)** and were analyzed by *β*-counting **(A)** or flow cytometry **(B, C)**. **(D, E)** Wt, *Jnk1^−/−^* **(D)** or *Jnk2^−/−^* **(E)** BMMCs were stimulated with IL-3. Cells were probed with [H^3^]thymidine and analyzed by *β*-counting. **(F, G)** BMMCs were pre-treated with SU6656 and were stimulated with IL-3. Cells were probed with [H^3^]thymidine and analyzed by *β*-counting **(F)** (*p* < 0,001) or lysates were analyzed by westernblotting **(G)**.

Next, we investigated whether the IKK-dependent JNK signaling is relevant for the IL-3-induced BMMC proliferation. The JNK inhibitor SP600125 significantly reduced the IL-3-induced proliferation (Figure 3A) without inducing cell death (Figure 3B and 3C). To determine which JNK isoform is relevant for the IL-3-induced proliferation we used *Jnk1^−/−^*-, *Jnk2^−/−^*- and *Jnk3^−/−^*-BMMCs. BMMCs do not express JNK3 (Supplementary Figure S2A–C). The surface expression of IL-3Rα is equal in wt, *Jnk1^−/−^*- and *Jnk2^−/−^*-BMMCs (Supplementary Figure S2D). *Jnk1^−/−^*-BMMC proliferated less strongly (Figure 3D) in response to IL-3 than wt- or *Jnk2^−/−^*-BMMCs (Figure 3E). Together these data indicate that the IL-3-induced proliferation depends on an IKK2-JNK1 signaling pathway.

### Subthreshold IKK activation is SFK-dependent and primes mast cells for NFκB-dependent effector functions

Next we investigated which pathway mediates subthreshold IKK activation. Given that the Malt/Bcl10-complex [32] and MyD88 (data not shown) are not involved we examined SFKs, critical for IKK2 activation and for mitogenic signaling [33–37]. The SFK inhibitor SU6656 blocked the IL-3-induced proliferation and inhibited the IL-3-induced JNK activation and IκBα phosphorylation (Figure 3F and 3G). In contrast, the IL-33-induced IKK activation was not affected by SU6656 (Supplementary Figure S3A) indicating that the SFK-dependent IKK activation is specific for the IL-3-induced signaling.

**Figure 4:**
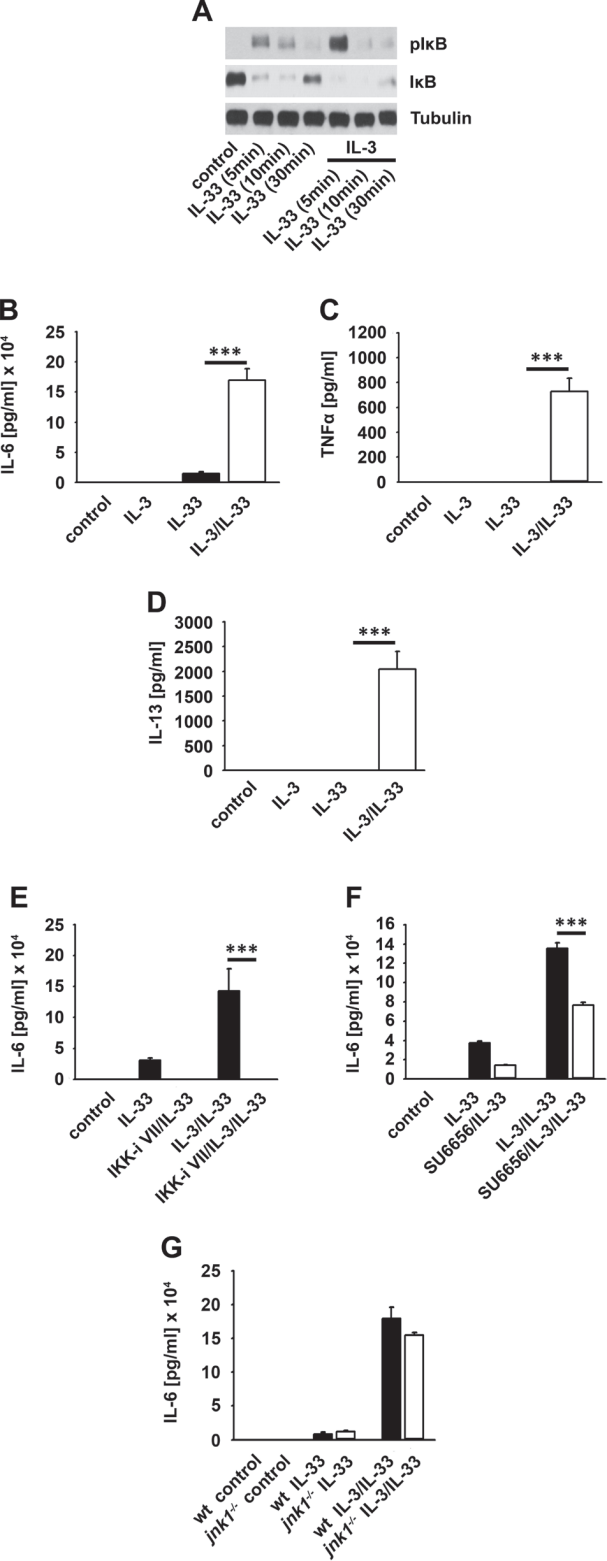
IL-3 primes BMMCs for stimulation with IL-33 **(A, B)** BMMCs were single stimulated with IL-33 or IL-33 in combination with IL-3. Lysates were analyzed by westernblotting **(A)** or collected supernatants were analyzed for IL-6 **(B)** (*p* < 0,001). **(C, D)** BMMCs were single stimulated with IL-33 or IL-33 in combination with IL-3. Supernatants were collected and analyzed for TNFα **(C)** (*p* < 0,001) or IL-13 **(D)** (*p* < 0,001). **(E, F)** BMMCs were pre-treated with the IKK-inhibitor VII **(E)** or SU6656 **(F)**. Cells were single stimulated with IL-33 or IL-33 in combination with IL-3. Collected supernatants were analyzed for IL-6 (**E**, **F**; *p* < 0,001). **(G)** Wt or *Jnk1^−/−^* BMMCs were single stimulated with IL-33 or IL-33 in combination with IL-3. Supernatants were collected and analyzed for IL-6.

SCF potentiates the IL-33-induced cytokine response in BMMCs [27]. Hence, we tested whether the IL-3-induced subthreshold IKK activation primes BMMCs for stronger NFκB activation upon IL-33R-signaling. Indeed, co-stimulation with IL-3 and IL-33 increased the IκBα phosphorylation, accelerated its degradation (Figure 4A) and potentiated the IL-6 mRNA production (Supplementary Figure S3B) compared to IL-33 alone. Consequently, IL-6 production after co-stimulation was much stronger than in response to IL-33 alone (Figure 4B). Notably, TNFα and IL-13 were only produced when BMMCs were co-stimulated with IL-3 and IL-33 but were not detectable upon stimulation with IL-33 alone (Figure 4C and 4D). Confirming the priming effect of IL-3, the full potentiated cytokine response was only detectable, when cells were first stimulated with IL-3 followed by exposure to IL-33. Pre-stimulation with IL-33 or simultaneous stimulation with IL-3 and IL-33 induced only a partial co-stimulatory effect (Supplementary Figure S3C).

**Figure 5:**
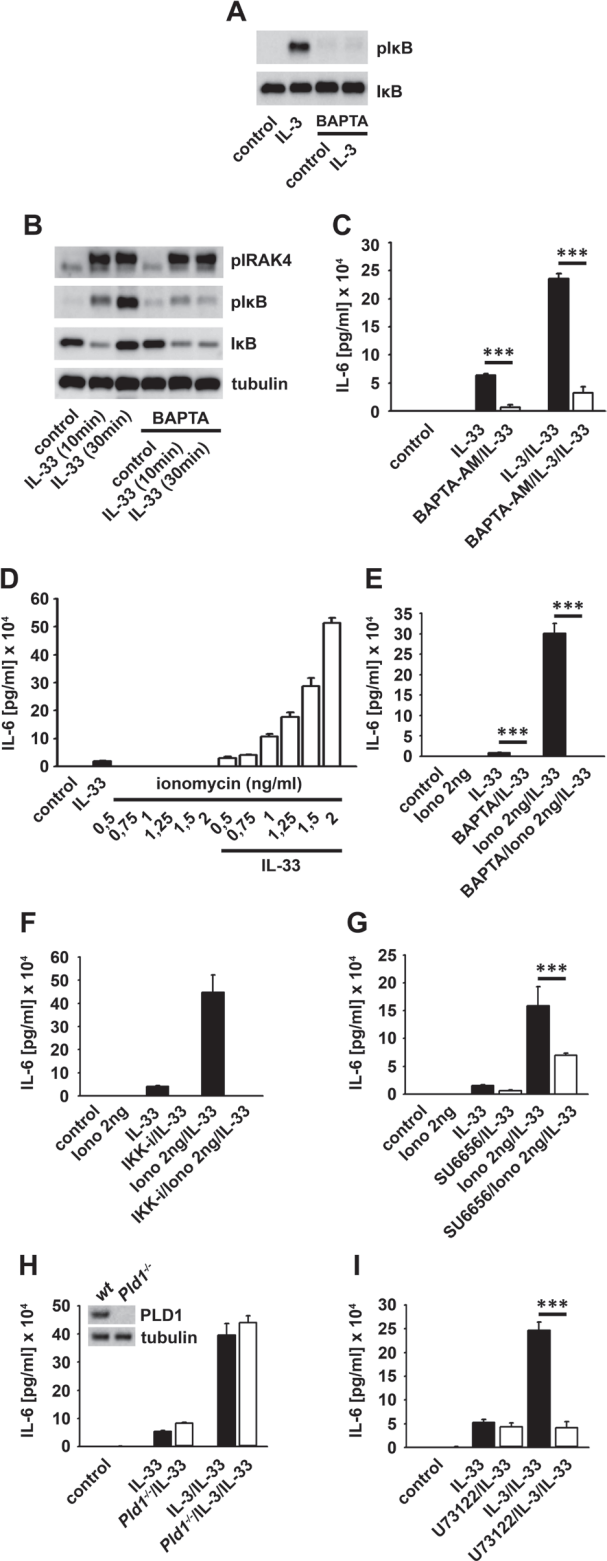
Ca^2+^ mediates the IL-3-induced mast cell priming **(A, B)** BMMCs were pre-treated with BAPTA-AM and stimulated with IL-3 **(A)** or IL-33 **(B)**. Lysates were analyzed by westernblotting. **(C)** BMMCs were pre-treated with BAPTA-AM and single stimulated with IL-33 or IL-33 in combination with IL-3. Supernatants were collected and analyzed for IL-6 (*p* < 0,001). **(D)** BMMCs were pre-treated with different ionomycin concentrations and stimulated with IL-33. Supernatants were collected and analyzed for IL-6. **(E–G)** BMMCs were pre-treated with BAPTA-AM **(E)** the IKK-inhibitor VII **(F)** or SU6656 **(G)** (E, G; *p* < 0,001). Cells were single stimulated with IL-33 or IL-33 in combination with ionomycin. Supernatants were collected and analyzed for IL-6. Wt, *Pld1^−/−^* **(H)** or U73122 **(I)** -treated BMMCs were single stimulated with IL-33 or IL-33 in combination with IL-3. Supernatants were collected and analyzed for IL-6 (**I**; *p* < 0,001).

**Figure 6:**
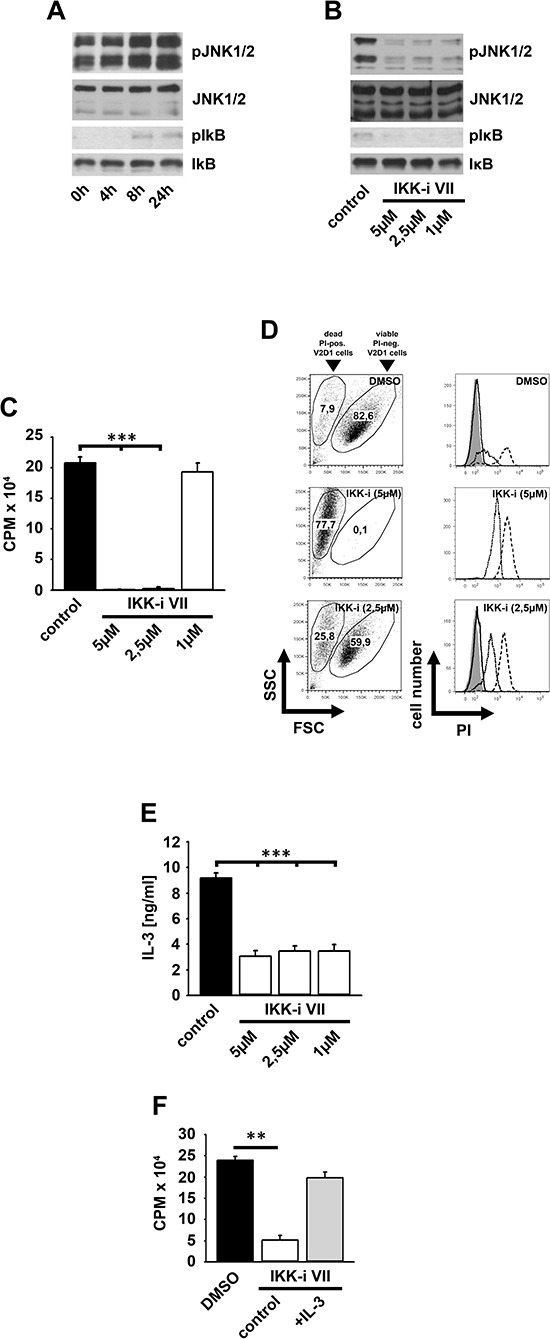
Survival of V2D1 cells depends on the IKK-mediated IL-3 production **(A)** V2D1 cells were cultured in IL-3-free medium for different time periods. Lysates were analyzed by westernblotting. **(B–D)** V2D1 cells were treated with the IKK-inhibitor VII. Lysates were analyzed by westernblotting **(B)** or cells were probed with [H^3^]thymidine **(C)** or PI **(D)**. Cells were analyzed by *β*-counting **(C)** or by flow cytometry **(D)** (**C**; *p* < 0,001). **(E)** V2D1 cells were treated with the IKK-inhibitor VII and collected supernatants were analyzed for IL-3 (*p* < 0,001). **(F)** V2D1 cells were treated with the IKK-inhibitor VII and stimulated with IL-3. Cells were probed with [H^3^]thymidine and were analyzed by *β*-counting (*p* < 0,01).

**Figure 7:**
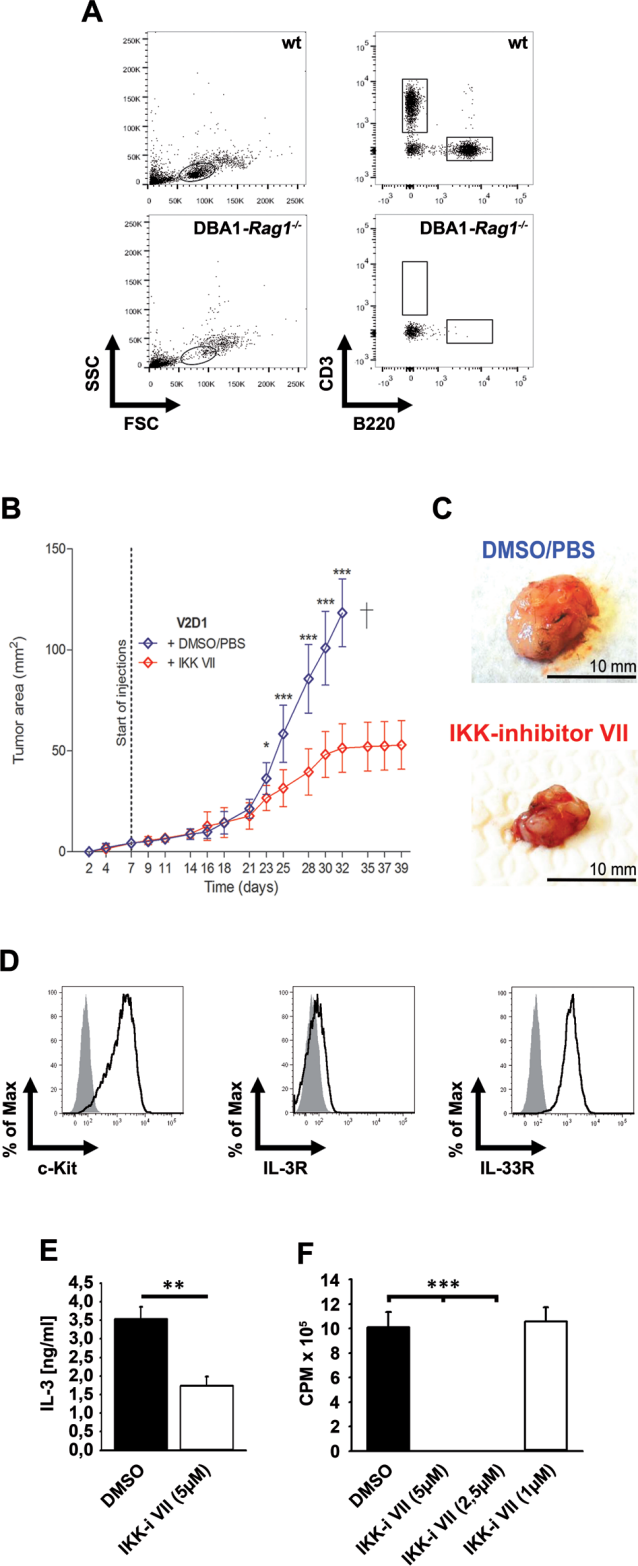
IKK inhibition reduced growth of V2D1 tumors **(A)** Blood from wt or DBA1-*Rag1^−/−^*-mice was analyzed for CD3 and B220. **(B)** V2D1-cells (1 × 10^6^) were injected subcutaneously into the flanks of DBA/1-*Rag1^−/−^*-mice and tumor area was assessed for 6 weeks using a Mitutoyo Quick Mini caliper. Growing tumors were either treated with DMSO/PBS (blue line) or with IKK-inhibitor VII (red line) (25 μM). [Data represent the mean SD of 10 mice with tumors (*p* < 0,001)]. **(C)** V2D1 tumor size after 5 weeks, (upper panel) treated with vehicle DMSO/PBS and (lower panel) treated with IKK-inhibitor VII. **(D)** Cells from an explanted tumor were analyzed for surface expression of c-Kit, IL-3Rα and IL-33R. **(E)** Explanted cells were left untreated or were treated with the IKK-inhibitor VII. Supernatants were collected and analyzed for IL-3 (*p* < 0,01). **(F)** Explanted cells were treated with the IKK-inhibitor VII, were probed with [H^3^]thymidine and analyzed by *β*-counting (*p* < 0,001).

Furthermore, cyclohexamide, MyD88-deficiency (Supplementary Figure S3D and S3E), the IKK-inhibitor VII (Figure 4E) and (5Z)-7-Oxozeaenol (TAK1 inhibitor) (data not shown) completely blocked the IL-33- and IL-3/IL-33-induced cytokine production. In contrast, the SFK-inhibitor SU6656 only reduced the cytokine production (Figure 4F) whereas JNK1-deficiency (Figure 4G) had no effect. These data show that the *de novo* synthesis of cytokines induced by IL-33 or IL-33 in combination with IL-3, strictly depends on the MyD88-TAK1-IKK2 signaling whereas SFKs have only modulatory functions. Thus, whereas the IL-3-induced and SFK-dependent JNK1 activation mediates proliferation it does not influence the IL-33-induced cytokine production.

### IL-3-induced mast cell priming also depends on Ca^2+^

Ca^2+^ mediates IL-3-inudced signaling [38] and is important for TNFα-induced IKK activation [37]. We investigated whether Ca^2+^ also mediates the IL-3-induced subthreshold IKK activation and consequently induces mast cell priming.

The Ca^2+^ chelator BAPTA-AM blocked the IL-3-induced IKK activation (Figure 5A), the IL-33-induced canonical NFκB signaling and cytokine production in BMMCs (Figure 5B and 5C) and NFκB-EGFP-MC/9 cells (Supplementary Figure S4A and S4B). This shows that Ca^2+^ is critical for the IL-3- and IL-33-induced IKK activation. Next we investigated the role of Ca^2+^ by using ionomycin. We used suboptimal ionomycin concentrations that neither induces NFAT activation nor cytokine production [39, 40] (Supplementary Figure S4C). Combined with IL-33, 2 ng ionomycin showed the most pronounced potentiated IL-6 production (Figure 5D) which was blocked by BAPTA-AM, the IKK-inhibitor VII and was reduced by SU6656 (Figure 5E–5G). Therefore, ionomycin mimics IL-3 stimulation and potentiates the IL-33-induced cytokine production.

Ca^2+^ mobilization induces NFAT activation. Thus, we tested whether stimulation with IL-33 alone or in combination with IL-3 induces NFAT activation. Neither IL-33 nor IL-33 in combination with IL-3 induced NFAT activation in NFAT-EGFP-MC/9 cells compared to ionomycin (Supplementary Figure S4D–F). These data show that Ca^2+^ is critical for the induced cytokine production independently of NFAT.

The Orai1/STIM1-Ca^2+^-channel system is important for FcεRI-mediated mast cell effector functions [41]. Therefore we hypothesized that the IL-33- and/or the IL-3/IL-33-induced cytokine production also depends on Orai1 and Stim1. Neither Orai1- (Supplementary Figure S4G) nor STIM1- (data not shown) deficiency affected the IL-33- or IL-3/IL-33-induced cytokine production.

The IL-3-induced Ca^2+^ mobilization depends on PLCγ [38]. In contrast, IL-33 does not induce PLCγ activation (data not shown) but was reported to induce a PLD1-dependent Ca^2+^ mobilization resulting in NFκB activation [42]. Neither treatment of NFκB-EGFP-MC/9 cells with the PLD1 inhibitor CAY10594 (Supplementary Figure S4H) nor PLD1 deficiency (Figure 5H) influenced NFκB activation or cytokine production. As expected the PLCγ inhibitor U-73122 did not affect the IL-33-induced cytokine response but blocked the potentiated cytokine response induced by co-stimulation with IL-3 and IL-33 (Figure 5I).

These data indicate that the IL-3-induced PLCγ activation and the resulting Ca^2+^ mobilization are crucial for mast cell priming and the resulting potentiated cytokine production.

### Subthreshold IKK activation mediates survival of tumor mast cells

In primary mast cells, IKK inactivation reduced the IL-3-induced mitogenic signaling. IL-3, c-Kit and IKKs are involved in pathogenesis of a number of malignancies [43–45]. Therefore, we determined the activation status of IKKs and their relevance for survival and proliferation in tumor mast cells. The v-HA-*Ras*-transformed V2D1 murine tumor mast cells constitutively produces IL-3, resulting in autocrine stimulation, proliferation and survival [46]. In these cells we found an increased JNK activation and IκBα phosphorylation but no degradation excluding the production of cytokines that activate NFκB (Figure 6A).

The IKK-inhibitor VII blocked the activation of JNKs, the phosphorylation of IκBα (Figure 6B) and consequently reduced cell proliferation (Figure 6C) by inducing cell death (Figure 6D). This demonstrates the relevance of IKKs in V2D1 cells. Given that survival of V2D1 cells depends on IL-3 production we speculated that IKKs are involved in IL-3 production and that exogenous IL-3 rescues V2D1 cells from cell death induced by IKK inhibition. As shown in Figure 6E IL-3 production is reduced by IKK inhibition. Moreover, the IKK-inhibitor VII-induced cell death can be reversed by exogenous IL-3 (Figure 6F). These data show that subthreshold IKK activation is important for IL-3 production to mediate mitogenic signaling in V2D1 cells.

### Inhibition of IKKs reduces tumor growth *in vivo*

To analyze tumor growth *in vivo*, we used DBA/1-*Rag1^−/−^*-mice (Figure 7A). We injected 1 × 10^6^ V2D1 mast cells subcutaneously. After 7 days, a 25 μM IKK-inhibitor VII solution or vehicle was injected intratumorally for 6 weeks. As shown in Figure 7B and 7C tumor size was significantly decreased in mice treated with the IKK-inhibitor. Cells obtained from an explanted tumor still expressed c-Kit, IL-3Rα, and IL-33R (Figure 7D) and showed IKK-dependent IL-3 production (Figure 7E) and proliferation (Figure 7F). These data verify that the explanted cancer cells are still dependent on IL-3 production for autocrine stimulation and survival.

## DISCUSSION

We identified IKKs as important for IL-3-induced mitogenic signaling in BMMCs. Hitherto, IKK2 has been known as an important component in the signaling pathways emanating from receptors such as antigen receptors, TIR- and TNFR-superfamily family members [32, 47]. In all of these cases, IKK2 activation results in IκBα degradation and NFκB activation [48]. In contrast to the canonical pathway, we found that IL-3 induced only a weak and transient subthreshold IKK activation, which resulted in IκBα phosphorylation without IκBα degradation and without NFκB activation.

The major questions are (i) how does IL-3 induce subthreshold IKK2 activation?; and (ii) why is there no IκBα degradation and NFκB activation? Neither MyD88- nor Malt1- or Bcl10-deficiency influence the IL-3-induced mast cell proliferation [32]. Instead, our data indicate a critical role of SFKs and Ca^2+^ for the IL-3-induced subthreshold IKK activation. We hypothesize that the combined activation of SFKs, PLCs and a Ca^2+^-dependent PKC-isoform mediates subthreshold IKK2 activation.

These data indicate that components (e.g., the MyD88-IRAK-TAK1-signaling module) critical for effective IκBα phosphorylation and degradation are not activated. Therefore the IL-3-induced IKK activation is only sufficient to induce mitogenic signaling, but is below the threshold to induce IκBα degradation and NFκB activation. Although the reason for the missing IκBα degradation is still unknown we suggest that IκBα degradation occurs to some extent upon IL-3 stimulation but is quantitatively not sufficient to induce a net loss of IκBα in the presence of ongoing IκBα re-synthesis.

Additionally, the IL-3-induced subthreshold IKK activation primes mast cells for enhanced NFκB activation in response to IL-33. This shows that mast cells integrate signals from different receptors which activate IKKs. Thereby, both, the IL-3-induced and SFK-mediated, and the IL-33-induced but MyD88-IRAK-TAK1-dependent pathways are crucial to facilitate full IKK2 activity.

Therefore the signal strength determines the effector functions resulting from IKK activation. Weak, subthreshold, IKK activation as induced by IL-3 suffices to induce proliferation but not NFκB activation. Stronger IKK activation as provided by IL-33 signaling results in NFκB activation and production of cytokines. Combined signaling via IL-3 and IL-33 results in proliferation and strongly enhanced cytokine production. Under homeostatic conditions IL-3 serves as growth and survival factor for mast cells. Under pathological conditions (tissue damage) the presence of the alarmin IL-33 dramatically alters the mast cells' response to IL-3 which now becomes much more pro-inflammatory (IL-6, TNFα, IL-13).

In tumor cells, dysregulated expression of IL-3 is the molecular basis for survival and proliferation [11, 43, 46, 49] leading to malignancies, including mast cell leukemia (MCL), acute myeloid leukemia (AML), and chronic myeloid leukemia (CML). In such cells subthreshold IKK activation might be a prerequisite for permanent proliferation. Indeed, pharmacological inhibition of subthreshold IKK activation specifically reduces tumor growth *in vivo*. Since IKK inhibition induced cell death in tumor- but not in primary-cells, IKK-inhibition could be a powerful approach to specifically eliminate certain tumor cells. In summary, our data indicate that the IL-3-induced subthreshold IKK activation is an important mechanism that mediates pathological effector functions in inflamed tissues after infection, allergy, necrosis and supports survival of tumor cells. Therefore, inhibition of subthreshold IKK activation might be a potential and selective tool to treat such mast cell driven diseases.

## METHODS

### Mice

Animal experiments were approved by the appropriate institutional and governmental committees for animal welfare. The xenograft model was performed in accordance with institutional guidelines on animal welfare and was approved by the “Landesamt für Natur, Umwelt und Verbraucherschutz” of Nordrhein-Westfalen (AZ 2011.A302). We used sex- and age-matched *Myd88^−/−^*, *Jnk1^−/−^* [50], *Jnk2^−/−^* [50, 51], *Jnk3^−/−^* [50, 52], *Ikk2^F/F^* [31], *Ikk2^Δ^* [31], *Pld1^−/−^* [53], *Orai1^−/−^* [54], *Stim1^−/−^* [41, 55], DBA1-*Rag1^−/−^*-mice and wild type (wt) littermates for generation of bone marrow-derived mast cells (BMMCs) or to perform the xenograft model.

### Cell culture

For BMMCs generation bone marrow was obtained from the femurs and tibias of mice. Bone marrow cells were cultured in IMDM (PAA) supplemented with 10% FCS, 100 U/ml penicillin, 100 mg/ml streptomycin, 50 mM 2-mercaptoethanol (complete medium) and 20 ng/ml mIL-3 (conditioned media from WEHI-3 cells). Mast cells were identified by expression of FcεRI, c-Kit, IL-33R and IL-3Rα by flow cytometry. The mast cell lines NFκB- and NFAT-EGFP-MC/9 (Dr. E. A. Barbu, Receptors and Signal Transduction Section, NIDCR, National Institutes of Health, Bethesda, Maryland, USA; [30]) were cultured in complete DMEM (PAA) supplemented with 20 ng/ml mIL-3 (conditioned media from WEHI-3 cells). V2D1-cells (Dr. Moroni, Department of Biochemistry, University of Basel, Switzerland; [46]) were cultured in complete IMDM (V2D1).

### Flow cytometry

Non-specific binding was blocked with anti-CD16/CD32 (clone 2.4G2) and rat-IgG (Jackson). Cells were stained with biotinylated anti-murine IL-33R antibody (3E10; [56]), PE-conjugated streptavidine, the APC-conjugated anti-CD123 antibody (R&D Systems), the PE-conjugated anti-murine CD117 antibody (Biolegend) or the anti-murine FcεRI antibody (FITC-conjugated) (eBioscience) (in PBS containing 0.25% BSA and 0.02% sodium azide).

For EGFP-production, NFκB- or NFAT-EGFP-MC/9 cells (10^6^ cells/ml) were IL-3-starved (1 h), pre-treated with inhibitors (30 min) and stimulated with IL-3 or IL-33 (8 h) (both Peprotech). After harvesting and washing (PBA-buffer; 0.25% BSA and 0.02% sodium azide in PBS) cells were analyzed by using an LSR II flow cytometer (BD) and FlowJo 8.1.1 (Treestar Inc.).

### Stimulation and lysis

BMMCs (10^6^ cells/ml) were IL-3-starved (1 h), pre-incubated (30 min) with SP600125 (JNK-inhibitor), SU6656 (SFK-inhibitor), IKK-inhibitors (VII, PS-1145, BMS-34554) and U73122 (PLC inhibitor) (If not other stated all these inhibitors were used in a concentration of 5 μM). Furthermore we used the protein biosynthesis inhibitor cyclohexamide (340 μM), the PLD1 inhibitor CAY10594 (10 μM), and the Ca^2+^ chelator BAPTA-AM (10 μM) or ionomycin (in a concentration from 0,5–10 ng/ml) (all inhibitors were from Merck Millipore). After pre-incubation of the indicated inhibitor cells were stimulated with IL-3 and/or IL-33 (Peprotec., in a concentration of 50 ng/ml). V2D1 cells were seeded (10^6^ cells/ml), pre-treated with the IKK-inhibitor VII (1 h). Cells were lysed (in 20 mM HEPES, pH7.5; 10 mM EGTA; 40 mM β-glycerophosphate; 2.5 mM MgCl_2_; 2 mM orthovanadate; 1 mM dithiothreitol; 20 μg/ml aprotinin; 20 μg/ml leupeptin, 1% Triton). Protein concentration was determined by using the BCA-assay (Pierce) and samples were boiled in 6 x Laemmli buffer.

### q PCR

Cells stimulated with IL-3 and/or IL-33 were pelleted and lysed with TRIzol (life technologies). RNA was extracted according to the manufacturer's protocol. Total RNA (2 μg) was reverse transcripted using oligo(dT)-primers, the M-MLV reverse transcriptase (affymetrix), RNase inhibitor (Promega) and the PCR thermocycler (Biometra). For the quantitative IL-6 real-time PCR the 5′ primer TCTCTGCAAGAGACTTCCATCCAGT and the 3′ primer AGCCTCCGACTTGTGAAGTGGT were used. The quantitative real-time PCR was performed with the KAPA SYBR fast kit (peqlab) according to the manufacturer's guidelines in an ABI Step OnePlus Real-Time PCR System (life technologies). GAPDH real-time PCR was performed by using the 5′ primer TTGGCCGTATTGGGCGCCTG and the 3′ primer CACCCTTCAAGTGGGCCCCG. Relative gene expression was determined using the conventional ßßCT method setting the control (unstimulated samples) as 1.

### Immunoblotting

Samples were separated on 10% sodium dodecyl sulfate (SDS)-Laemmli gels and transferred by electroblotting onto nitrocellulose membranes (Biostep). Membranes were blocked in 0.1% Tween/TBS-buffer with 5% dry milk and incubated with antibodies detecting phosphorylated/non-phosphorylated proteins. We used anti-pT183/Y185-JNK-1/2/JNK-1/2, anti-pS176/177-IKK1/2/IKK1/2, anti-pT345/S346-IRAK4, anti-pS32-IκBα/IκBα, anti-PLD1 and anti-tubulin (Cell Signaling; except anti-IKK1/2 from Santa Cruz and anti-tubulin from Sigma). Membranes were washed in 0.1% Tween/TBS and incubated with HRP-conjugated secondary antibodies: anti-rabbit-Ig, anti-goat-Ig (Santa Cruz) and anti-mouse-Ig (Thermo). Detection was performed using ECL reagent (Pierce).

### Cell death and cell counting

10^6^ cells/ml (BMMCs) or 10^5^ cells/ml (V2D1) were pre-treated with the IKK-inhibitor VII or SP600125 (48 h), harvested, washed (PBA-buffer) and treated with propidium iodide (PI). Cells were analyzed with the LSR II flow cytometer (BD) and FlowJo 8.1.1 (Treestar Inc.).

For cell counting BMMCs (10^6^ cells/ml) were seeded in IL-3-free media. After 1 h cells were treated with IKK-inhibitor VII (for 30 min) and were stimulated with IL-3 as indicated. Cells were 1:1 mixed with trypan blue solution and trypan blue-negative cells were counted by using Neubauer counting chamber.

### ELISA and proliferation assays

BMMCs (10^6^ cells/ml) or V2D1 (10^5^ cells/ml) were seeded in IL-3-free media. Cells were incubated with vehicle (DMSO) or inhibitors. BMMC were stimulated with IL-3, IL-33 or both (Peprotech). Supernatants were analyzed for IL-3, IL-6, TNFα and IL-13 using matched pair antibodies (eBioscience) by ELISA. For proliferation assays cells were cultured for 54 h. [H^3^]thymidin (1 μCi) was added for additional 18 h. Incorporated radioactivity was measured by using a *β*-scintillation counter (Perkin Elmer). The increase of mast cell proliferation is indicated as the stimulation index (SI). Thereby the basal CPM values were set to 1.

### Characterization of DBA/1-*Rag1*^−/−^-mice

Blood was taken from facial vein from 4–6 week old mice. After lysis of erythrocytes, samples were washed in PBA, stained with anti-CD3 (clone 145–2C11, ebioscience, Germany) and anti-B220 (clone RA3–6B2) and analyzed with the LSR II flow cytometer (BD, USA) and FlowJo (Treestar Inc., USA).

### Xenograft tumor model

DBA/1-*Rag1^−/−^*-mice (10–18 weeks old) received 10^6^ V2D1-cells in 100 μl subcutaneously into the shaved flanks. Animals were treated 3 times weekly with vehicle (1% DMSO/PBS) or the IKK-inhibitor VII (100 μl; 25 μM in 1% DMSO/PBS) subcutaneously around the tumors. Tumor size was assessed 3 times weekly by using a Mitutoyo Quick Mini caliper (Mitutoyo). Measurements were recorded as tumor area (mm^2^) from groups of 10 mice. Tumor area (A) was calculated by the formula: [A = tumor height x tumor width]. Experiments were terminated after 6 weeks or if tumor size exceeded the ethically approved dimensions.

### Statistics

All experiments were performed at least three times (shown is one representative experiment). Proliferation assays, and ELISAs were performed three times in at least a 6-fold determination. Cytokine concentration is indicated as the mean of measurements ± standard deviation. For proliferation assays and ELISA one representative experiment is shown. The statistical analysis was performed with IBM SPSS Statistics version 20.0 (IBM). Statistical significance was assessed by Mann-Whitney-U test. Statistical significance was accepted for *p* < 0,05 (*,*p* < 0.05; **,*p* < 0.01; ***,*p* < 0.001).

## SUPPLEMENTARY FIGURES



## References

[1] St John AL, Abraham SN (2013). Innate immunity and its regulation by mast cells. J Immunol.

[2] Malaviya R, Ikeda T, Ross E, Abraham SN (1996). Mast cell modulation of neutrophil influx and bacterial clearance at sites of infection through TNF-alpha. Nature.

[3] Shelburne CP, Nakano H, St John AL, Chan C, McLachlan JB, Gunn MD, Staats HF, Abraham SN (2009). Mast cells augment adaptive immunity by orchestrating dendritic cell trafficking through infected tissues. Cell host & microbe.

[4] Shin K, Watts GF, Oettgen HC, Friend DS, Pemberton AD, Gurish MF, Lee DM (2008). Mouse mast cell tryptase mMCP-6 is a critical link between adaptive and innate immunity in the chronic phase of Trichinella spiralis infection. J Immunol.

[5] Orinska Z, Bulanova E, Budagian V, Metz M, Maurer M, Bulfone-Paus S (2005). TLR3-induced activation of mast cells modulates CD8+ T-cell recruitment. Blood.

[6] Dudeck A, Dudeck J, Scholten J, Petzold A, Surianarayanan S, Kohler A, Peschke K, Vohringer D, Waskow C, Krieg T, Muller W, Waisman A, Hartmann K, Gunzer M, Roers A (2011). Mast cells are key promoters of contact allergy that mediate the adjuvant effects of haptens. Immunity.

[7] Lee DM, Friend DS, Gurish MF, Benoist C, Mathis D, Brenner MB (2002). Mast cells: a cellular link between autoantibodies and inflammatory arthritis. Science.

[8] Feyerabend TB, Weiser A, Tietz A, Stassen M, Harris N, Kopf M, Radermacher P, Moller P, Benoist C, Mathis D, Fehling HJ, Rodewald HR (2011). Cre-mediated cell ablation contests mast cell contribution in models of antibody- and T cell-mediated autoimmunity. Immunity.

[9] Guma M, Kashiwakura J, Crain B, Kawakami Y, Beutler B, Firestein GS, Kawakami T, Karin M, Corr M (2010). JNK1 controls mast cell degranulation and IL-1{beta} production in inflammatory arthritis. Proc Natl Acad Sci U S A.

[10] Rabenhorst A, Schlaak M, Heukamp LC, Forster A, Theurich S, von Bergwelt-Baildon M, Buttner R, Kurschat P, Mauch C, Roers A, Hartmann K (2012). Mast cells play a protumorigenic role in primary cutaneous lymphoma. Blood.

[11] Nair AP, Hirsch HH, Moroni C (1992). Mast cells sensitive to v-H-ras transformation are hyperinducible for interleukin 3 expression and have lost tumor-suppressor activity. Oncogene.

[12] Nagata H, Worobec AS, Oh CK, Chowdhury BA, Tannenbaum S, Suzuki Y, Metcalfe DD (1995). Identification of a point mutation in the catalytic domain of the protooncogene c-kit in peripheral blood mononuclear cells of patients who have mastocytosis with an associated hematologic disorder. Proc Natl Acad Sci U S A.

[13] Marone G, Casolaro V, Patella V, Florio G, Triggiani M (1997). Molecular and cellular biology of mast cells and basophils. International archives of allergy and immunology.

[14] Moritz DR, Rodewald HR, Gheyselinck J, Klemenz R (1998). The IL-1 receptor-related T1 antigen is expressed on immature and mature mast cells and on fetal blood mast cell progenitors. J Immunol.

[15] Ho LH, Ohno T, Oboki K, Kajiwara N, Suto H, Iikura M, Okayama Y, Akira S, Saito H, Galli SJ, Nakae S (2007). IL-33 induces IL-13 production by mouse mast cells independently of IgE-FcepsilonRI signals. Journal of leukocyte biology.

[16] Schmitz J, Owyang A, Oldham E, Song Y, Murphy E, McClanahan TK, Zurawski G, Moshrefi M, Qin J, Li X, Gorman DM, Bazan JF, Kastelein RA (2005). IL-33, an interleukin-1-like cytokine that signals via the IL-1 receptor-related protein ST2 and induces T helper type 2-associated cytokines. Immunity.

[17] Ma P, Vemula S, Munugalavadla V, Chen J, Sims E, Borneo J, Kondo T, Ramdas B, Mali RS, Li S, Hashino E, Takemoto C, Kapur R (2011). Balanced interactions between Lyn, the p85alpha regulatory subunit of class I(A) phosphatidylinositol-3-kinase, and SHIP are essential for mast cell growth and maturation. Molecular and cellular biology.

[18] Duttlinger R, Manova K, Chu TY, Gyssler C, Zelenetz AD, Bachvarova RF, Besmer P (1993). W-sash affects positive and negative elements controlling c-kit expression: ectopic c-kit expression at sites of kit-ligand expression affects melanogenesis. Development.

[19] Grimbaldeston MA, Chen CC, Piliponsky AM, Tsai M, Tam SY, Galli SJ (2005). Mast cell-deficient W-sash c-kit mutant Kit W-sh/W-sh mice as a model for investigating mast cell biology *in vivo*. The American journal of pathology.

[20] Yu WM, Hawley TS, Hawley RG, Qu CK (2002). Role of the docking protein Gab2 in beta(1)-integrin signaling pathway-mediated hematopoietic cell adhesion and migration. Blood.

[21] Wheadon H, Edmead C, Welham MJ (2003). Regulation of interleukin-3-induced substrate phosphorylation and cell survival by SHP-2. The Biochemical journal.

[22] Fukao T, Yamada T, Tanabe M, Terauchi Y, Ota T, Takayama T, Asano T, Takeuchi T, Kadowaki T, Hata Ji J, Koyasu S (2002). Selective loss of gastrointestinal mast cells and impaired immunity in PIK-deficient mice. Nat Immunol.

[23] Ali K, Bilancio A, Thomas M, Pearce W, Gilfillan AM, Tkaczyk C, Kuehn N, Gray A, Giddings J, Peskett E, Fox R, Bruce I, Walker C, Sawyer C, Okkenhaug K, Finan P (2004). Essential role for the p110delta phosphoinositide 3-kinase in the allergic response. Nature.

[24] Yu M, Luo J, Yang W, Wang Y, Mizuki M, Kanakura Y, Besmer P, Neel BG, Gu H (2006). The scaffolding adapter Gab2, via Shp-2, regulates kit-evoked mast cell proliferation by activating the Rac/JNK pathway. J Biol Chem.

[25] Saluja R, Delin I, Nilsson GP, Adner M (2012). FcepsilonR1-mediated mast cell reactivity is amplified through prolonged Toll-like receptor-ligand treatment. PLoS One.

[26] Andrade MV, Iwaki S, Ropert C, Gazzinelli RT, Cunha-Melo JR, Beaven MA (2011). Amplification of cytokine production through synergistic activation of NFAT and AP-1 following stimulation of mast cells with antigen and IL-33. European journal of immunology.

[27] Drube S, Heink S, Walter S, Lohn T, Grusser M, Gerbaulet A, Berod L, Schons J, Dudeck A, Freitag J, Grotha S, Reich D, Rudeschko O, Norgauer J, Hartmann K, Roers A (2010). The receptor tyrosine kinase c-Kit controls IL-33 receptor signaling in mast cells. Blood.

[28] Drube S, Schmitz F, Gopfert C, Weber F, Kamradt T (2012). C-Kit controls IL-1beta-induced effector functions in HMC-cells. European journal of pharmacology.

[29] Sandow JJ, Jabbour AM, Condina MR, Daunt CP, Stomski FC, Green BD, Riffkin CD, Hoffmann P, Guthridge MA, Silke J, Lopez AF, Ekert PG (2012). Cytokine receptor signaling activates an IKK-dependent phosphorylation of PUMA to prevent cell death. Cell death and differentiation.

[30] Barbu EA, Zhang J, Siraganian RP (2010). The limited contribution of Fyn and Gab2 to the high affinity IgE receptor signaling in mast cells. J Biol Chem.

[31] Mankan AK, Canli O, Schwitalla S, Ziegler P, Tschopp J, Korn T, Greten FR (2011). TNF-alpha-dependent loss of IKKbeta-deficient myeloid progenitors triggers a cytokine loop culminating in granulocytosis. Proc Natl Acad Sci U S A.

[32] Klemm S, Gutermuth J, Hultner L, Sparwasser T, Behrendt H, Peschel C, Mak TW, Jakob T, Ruland J (2006). The Bcl10-Malt1 complex segregates Fc epsilon RI-mediated nuclear factor kappa B activation and cytokine production from mast cell degranulation. J Exp Med.

[33] Samayawardhena LA, Kapur R, Craig AW (2007). Involvement of Fyn kinase in Kit and integrin-mediated Rac activation, cytoskeletal reorganization, and chemotaxis of mast cells. Blood.

[34] Samayawardhena LA, Pallen CJ (2010). PTPalpha activates Lyn and Fyn and suppresses Hck to negatively regulate FcepsilonRI-dependent mast cell activation and allergic responses. J Immunol.

[35] Lee JH, Kim JW, Kim do Kim K, Park HS, Park HJ, Kim DK, Kim AR, Beaven B, Park MA, Kim KL, Choi YM (2011). The Src family kinase Fgr is critical for activation of mast cells and IgE-mediated anaphylaxis in mice. J Immunol.

[36] Hong H, Kitaura J, Xiao W, Horejsi V, Ra C, Lowell CA, Kawakami Y, Kawakami T (2007). The Src family kinase Hck regulates mast cell activation by suppressing an inhibitory Src family kinase Lyn. Blood.

[37] Huang WC, Chen JJ, Inoue H, Chen CC (2003). Tyrosine phosphorylation of I-kappa B kinase alpha/beta by protein kinase C-dependent c-Src activation is involved in TNF-alpha-induced cyclooxygenase-2 expression. J Immunol.

[38] Hidano S, Kitamura D, Kumar L, Geha RS, Goitsuka R (2012). SLP-76 is required for high-affinity IgE receptor- and IL-3 receptor-mediated activation of basophils. Int Immunol.

[39] Hsu CL, Bryce PJ (2012). Inducible IL-33 expression by mast cells is regulated by a calcium-dependent pathway. J Immunol.

[40] Hsu CL, Neilsen CV, Bryce PJ (2010). IL-33 is produced by mast cells and regulates IgE-dependent inflammation. PLoS One.

[41] Baba Y, Nishida K, Fujii Y, Hirano T, Hikida M, Kurosaki T (2008). Essential function for the calcium sensor STIM1 in mast cell activation and anaphylactic responses. Nat Immunol.

[42] Pushparaj PN, Tay HK, H'Ng SC, Pitman N, Xu D, McKenzie A, Liew FY, Melendez AJ (2009). The cytokine interleukin-33 mediates anaphylactic shock. Proc Natl Acad Sci U S A.

[43] Dorsey JF, Cunnick JM, Lanehart R, Huang M, Kraker AJ, Bhalla KN, Jove R, Wu J (2002). Interleukin-3 protects Bcr-Abl-transformed hematopoietic progenitor cells from apoptosis induced by Bcr-Abl tyrosine kinase inhibitors. Leukemia.

[44] Jiang X, Lopez A, Holyoake T, Eaves A, Eaves C (1999). Autocrine production and action of IL-3 and granulocyte colony-stimulating factor in chronic myeloid leukemia. Proc Natl Acad Sci U S A.

[45] Sharma N, Everingham S, Zeng LF, Zhang ZY, Kapur R, Craig AW (2014). Oncogenic KIT-induced aggressive systemic mastocytosis requires SHP2/PTPN11 phosphatase for disease progression in mice. Oncotarget.

[46] Nair AP, Diamantis ID, Conscience JF, Kindler V, Hofer P, Moroni C (1989). A v-H-ras-dependent hemopoietic tumor model involving progression from a clonal stage of transformation competence to autocrine interleukin 3 production. Molecular and cellular biology.

[47] Lin X, Wang D (2004). The roles of CARMA1, Bcl10, and MALT1 in antigen receptor signaling. Seminars in immunology.

[48] Kawai T, Akira S (2007). Signaling to NF-kappaB by Toll-like receptors. Trends in molecular medicine.

[49] Nair AP, Moroni C (1987). v-H-ras gene reduces IL-3 requirement in PB-3c mastocytes *in vitro* followed by autokrine tumor formation *in vivo*. Haematology and blood transfusion.

[50] Reinecke K, Herdegen T, Eminel S, Aldenhoff JB, Schiffelholz T (2013). Knockout of c-Jun N-terminal kinases 1, 2 or 3 isoforms induces behavioural changes. Behavioural brain research.

[51] Sabapathy K, Hu Y, Kallunki T, Schreiber M, David JP, Jochum W, Wagner EF, Karin M (1999). JNK2 is required for efficient T-cell activation and apoptosis but not for normal lymphocyte development. Current biology : CB.

[52] Yang DD, Kuan CY, Whitmarsh AJ, Rincon M, Zheng TS, Davis RJ, Rakic P, Flavell RA (1997). Absence of excitotoxicity-induced apoptosis in the hippocampus of mice lacking the Jnk3 gene. Nature.

[53] Elvers M, Stegner D, Hagedorn I, Kleinschnitz C, Braun A, Kuijpers ME, Boesl M, Chen Q, Heemskerk JW, Stoll G, Frohman MA, Nieswandt B (2010). Impaired alpha(IIb)beta integrin activation and shear-dependent thrombus formation in mice lacking phospholipase D1. Sci Signal.

[54] Braun A, Gessner JE, Varga-Szabo D, Syed SN, Konrad S, Stegner D, Vogtle T, Schmidt RE, Nieswandt B (2009). STIM1 is essential for Fcgamma receptor activation and autoimmune inflammation. Blood.

[55] Varga-Szabo D, Braun A, Kleinschnitz C, Bender M, Pleines I, Pham M, Renne T, Stoll G, Nieswandt B (2008). The calcium sensor STIM1 is an essential mediator of arterial thrombosis and ischemic brain infarction. J Exp Med.

[56] Lohning M, Stroehmann A, Coyle AJ, Grogan JL, Lin S, Gutierrez-Ramos JC, Levinson D, Radbruch A, Kamradt T (1998). T1/ST2 is preferentially expressed on murine Th2 cells, independent of interleukin 4, interleukin 5, and interleukin 10, and important for Th2 effector function. Proc Natl Acad Sci U S A.

