# Supplementary material for: Subthreshold IKK activation modulates the effector functions of primary mast cells and allows specific targeting of transformed mast cells
Source: Oncotarget. 2015 Feb 27;6(7):5354–68. doi: 10.18632/oncotarget.3022 (PMC4467154; doi:10.18632/oncotarget.3022)
Supplement: Supplementary file 1 [file oncotarget-06-5354-s001.pdf]

SUPPLEMENTARY FIGURES

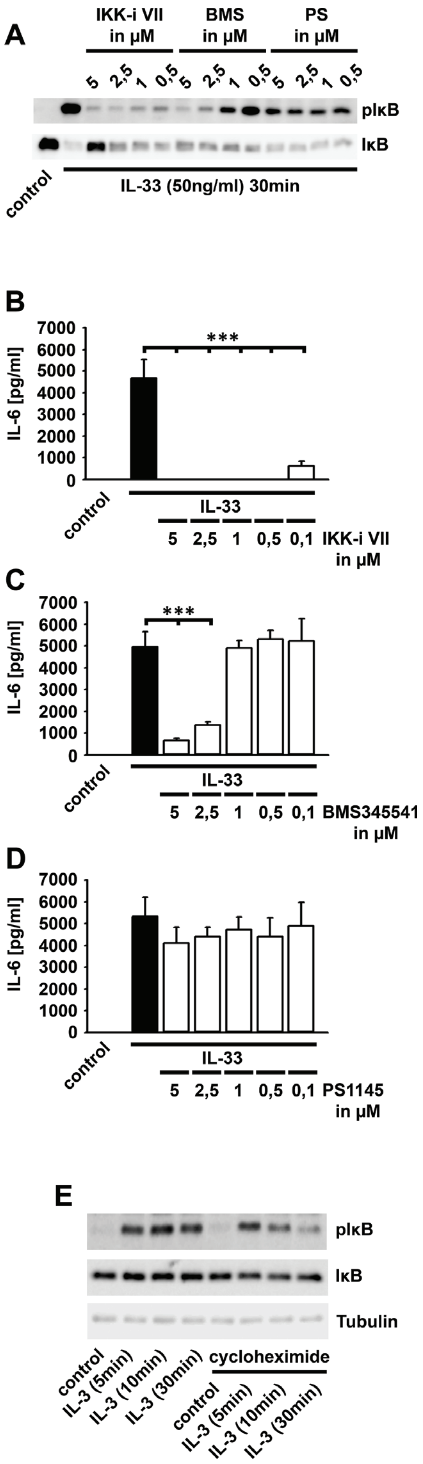

Supplementary Figure S1: The IKK inhibitor VII is the most potent IKK inhibitor. **(A–D)** BMMCs were pre-treated with different IKK inhibitors (as indicated) and stimulated with IL-33. Lysates were analyzed by westernblotting **(A)** or supernatants were collected and analyzed for IL-6 **(B–D)** (p < 0,001). **(E)** BMMCs were treated with cyclohexamide (340 µM) and stimulated with IL-3. Lysates were analyzed by westernblotting.

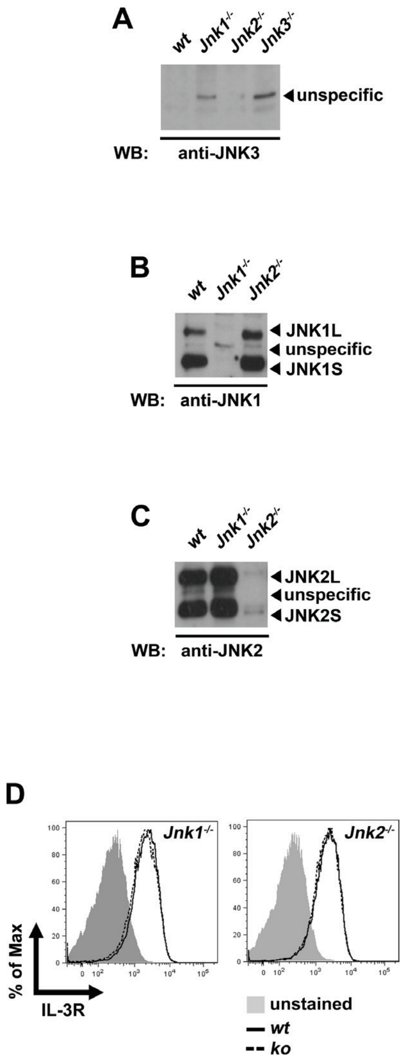

Supplementary Figure S2: IKK2 mediates JNK-dependent mitogenic signaling. (A–C) *Jnk1*$^{-/-}$, *Jnk2*$^{-/-}$ and *Jnk3*$^{-/-}$ BMMCs were lysed and analyzed for the expression of JNK isoforms by westernblotting. **(D)** *Jnk1*$^{-/-}$ and *Jnk2*$^{-/-}$ BMMCs were analyzed for IL-3Rα expression by flow cytometry.

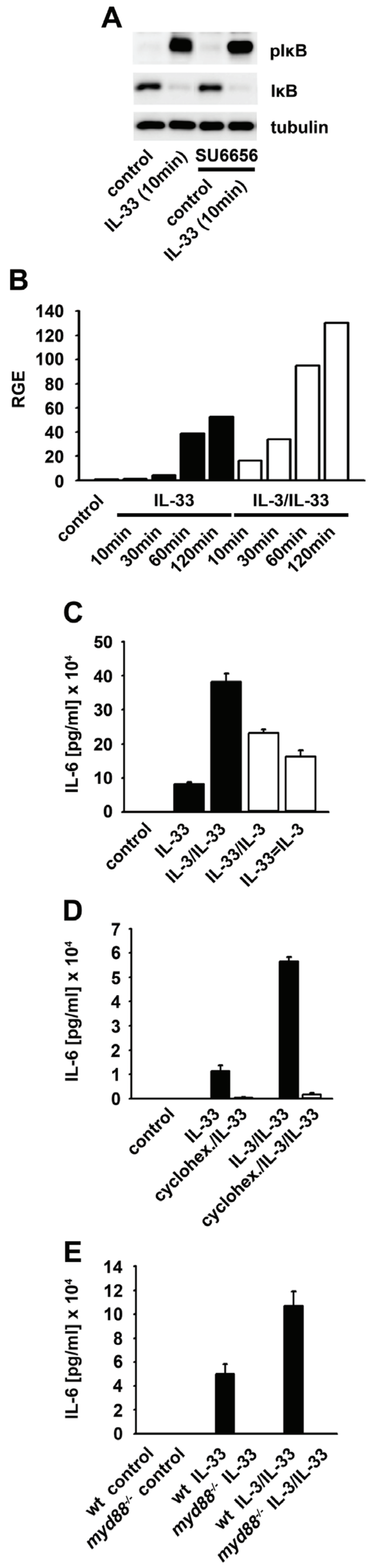

Supplementary Figure S3: The cytokine production depends on *de novo* protein-biosynthesis. (A) BMMCs were pre-treated with SU6656 and stimulated with IL-33. Lysates were analyzed by westernblotting. **(B)** BMMCs were single stimulated with IL-33 or IL-33 in combination with IL-3. Total RNA was isolated, reverse transcribed and analyzed for the presence of IL-6 mRNA. **(C)** BMMCs were single stimulated with IL-33 or were sequentially (pre-stimulation with IL-3 for 30min: IL-3/IL-33 or pre-stimulation with IL-33: IL-33/IL-3) or simultaneously (IL-33=IL-3) stimulated. Supernatants were collected and analyzed for IL-6. **(D, E)** Cycloheximide-treated- **(D)**, wt or $Myd88^{-/-}$ **(E)** BMMCs were single stimulated with IL-33 or IL-33 in combination with IL-3. Supernatants were collected and analyzed for IL-6.

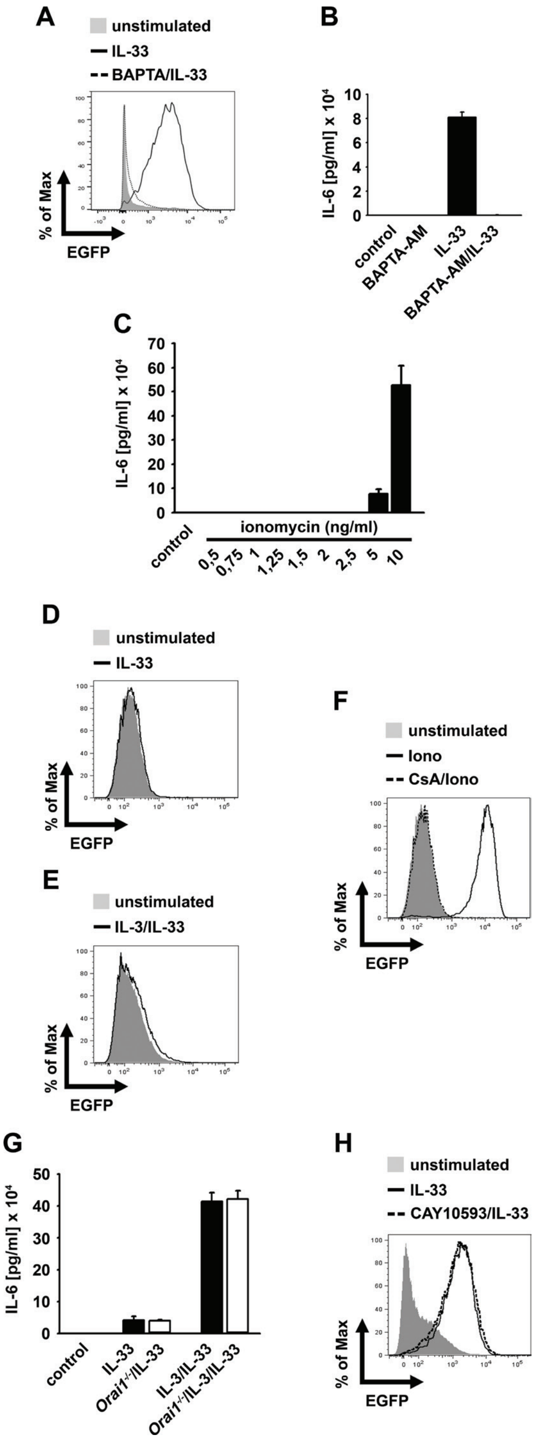

Supplementary Figure S4: The IL-33- or IL-3/IL-33-induced cytokine production depends on Ca^{2+}. (A, B) NFκB-EGFP-MC/9 cells were pre-incubated with BAPTA-AM and stimulated with IL-33. Cells were analyzed for EGFP-production by flow cytometry **(A)** or supernatants were collected and analyzed for IL-6 **(B)**. **(C)** BMMCs were treated with different ionomycin concentrations. Supernatants were collected and analyzed for IL-6 production. **(D–F)** NFκB-EGFP-MC/9 cells were stimulated with IL-33, **(D)** IL-33 in combination with IL-3 **(E)** or were pre-treated with BAPTA-AM and stimulated with ionomycin **(F)**. Cells were analyzed for EGFP production by flow cytometry **(D–F)**. **(G)** Wt or *Orai1*$^{-/-}$ BMMCs were stimulated with IL-33 or IL-33 in combination with IL-3. Supernatants were collected and analyzed for IL-6. **(H)** NFκB-EGFP-MC/9 were pre-treated with the PLD1 inhibitor Cay10593 and stimulated with IL-33. Supernatant were analyzed for IL-6.
